# Parents' Views on Play and the Goal of Early Childhood Education in Relation to Children's Home Activity and Executive Functions: A Cross-Cultural Investigation

**DOI:** 10.3389/fpsyg.2021.646074

**Published:** 2021-04-26

**Authors:** Biruk K. Metaferia, Judit Futo, Zsofia K. Takacs

**Affiliations:** ^1^Doctoral School of Psychology, ELTE Eötvös Loránd University, Budapest, Hungary; ^2^Neuropsychology and Child Development Research Group, ELTE Eötvös Loránd University, Budapest, Hungary; ^3^Institute of Psychology, ELTE Eötvös Loránd University, Budapest, Hungary; ^4^Institute of Education, ELTE Eötvös Loránd University, Budapest, Hungary

**Keywords:** executive function, home experience, parental view, preschool children, play

## Abstract

The present study investigated the cross-cultural variations in parents' views on the role of play in child development and the primary purpose of preschool education from Ethiopia and Hungary. It also examined the cross-cultural variations in preschoolers' executive functions (EF), the frequency of their engagement in home activities, and the role of these activities in the development of EF skills. Participants included 266 preschoolers (Ethiopia: 139 of which 44.6% boys; *M*_age_ = 63.83 months; *SD* = 7.68 months; Hungary: 127 of which 48% boys; *M*_age_ = 62.06 months; *SD* = 9.37 months) with their parents (Ethiopia: 45.32% male; M_age_ = 36.66 years; *SD* = 7.14 years; Hungary: 13.18% male; *M*_age_ = 37.71 years; *SD* = 5.97 years). The independent samples *t*-test showed that Ethiopian parents view fostering academic skills for preschooler significantly more important than their Hungarian counterparts do. We also found that while Ethiopian parents hold the belief that academic and cognitive development is the major purpose of preschool education, Hungarian prioritize social-emotional development and entertainment. Additionally, preschoolers in Ethiopia were reported to engage in academic and arts and crafts activities after preschool significantly more frequently than their Hungarian counterparts. On the contrary, preschoolers in Hungary were found to engage in fine-motor activities, solitary play, sports and other physical activities significantly more frequently than their Ethiopian counterparts. No significant differences were found in EF skills between preschoolers from Ethiopia and Hungary. Results from hierarchical regression analyses showed that, after accounting for age and SES, preschoolers' frequency of pretend play and their parents' play support beliefs were found to be small to medium-sized predictors of inhibitory control skills in both samples. However, children's frequency of having breakfast at home was another significant predictor in the Ethiopian sample only. The frequency of participation in arts and crafts and other fine-motor activities were found to be important predictors of preschoolers' visual-spatial working memory skills in the Ethiopian and the Hungarian samples, respectively. We also found that, after controlling for SES, parental play support was an important factor associated with preschoolers' shifting skills only in the Hungarian sample. Based on the findings, we made important conclusions.

## Introduction

The preschool years have been recognized as an important period when the development of executive function (EF) skills undergoes marked improvements (Diamond et al., 1997; Carlson, 2005; Garon et al., 2008; Best and Miller, 2010; Welsh et al., 2010; Anderson and Reidy, 2012; Howard et al., 2015; Nieto et al., 2016). The successful development of preschoolers' EF has been linked with better school adjustment and academic success in the beginning of primary education (Blair and Raver, 2015; Moriguchi et al., 2016). On the other hand, inadequate development of EF skills is linked with different physical, psychological, and academic-related problems such as physical aggression (Séguin and Zelazo, 2005), developmental psychopathology (Pennington and Ozonoff, 1996), and school readiness and difficulties in academic progress (Blair, 2002; Diamond, 2007). The present study investigated the cross-cultural variation in these important skills in preschoolers and the contribution of preschoolers' home experience and parental play beliefs in the development of EF skills.

EF refers to a set of cognitive skills responsible for the conscious control and regulation of one's thoughts and actions to guide goal-directed thinking and action (Diamond, 2013; Zelazo et al., 2016). It is generally considered to consist of three independent but interrelated components. Whether the same applies in childhood remains a question. For instance, studies by Fuhs and Day (2011) and Wiebe et al. (2011) with preschoolers support the unidimensional nature of EF in childhood. On the contrary, there are studies evidencing the different components of EF in the early period of life (see Usai et al., 2014; Howard et al., 2015; Skogan et al., 2015). In this line, previous studies (such as Miyake et al., 2000; Diamond, 2013; Howard et al., 2015; Zelazo et al., 2016) found that in the preschool years EF skills are composed of three core competencies: working memory, inhibition, and cognitive flexibility.

Working memory refers to the ability to concurrently hold information in mind and operate with it for a brief period of time (Baddeley and Hitch, 1974; Baddeley, 1992). According to Lan et al. (2011), working memory is regarded as an important element of EF since children must conceive and temporarily store information when they successfully perform a task. Based on its content, working memory can be categorized as verbal/semantic and non-verbal/visuospatial working memory (Miyake et al., 2000; Diamond, 2013).

Inhibition is defined as the cognitive ability to regulate one's thoughts and actions in order to dominate a strong internal tendency and/or external temptation (Diamond, 2013). It helps a person to get all the facts before reaching a conclusion (Montoya et al., 2018).

Cognitive flexibility is the capacity to flexibly switch from one mindset to another in order to adapt one's course of thoughts and action to a changing situation (Davidson et al., 2006; Garon et al., 2008). For instance, cognitive flexibility is needed when we shift our attention from one dimension/feature of a task to another (Miyake et al., 2000; Diamond, 2013).

The association between EF and early literacy skills is well-documented (Bierman et al., 2008; Foy and Mann, 2013; Weiland et al., 2014; Davidse et al., 2015; Lonigan et al., 2016). There have also been many compelling studies that prove the relationship between EF and different elements of school readiness that include competencies in language, math, science in addition to social-emotion skills (Carlson and Wang, 2007; Bull et al., 2008; Ponitz et al., 2009; Nayfeld et al., 2013). There is also extensive evidence indicating the predictive (both concurrent and longitudinal) power of EF in literacy and reading (Adams and Snowling, 2001; Welsh et al., 2010), mathematics (Bull and Scerif, 2001; St Clair-Thompson and Gathercole, 2006; Brock et al., 2009; Lee et al., 2012) and short- and long-term academic success (Blair and Razza, 2007; McClelland et al., 2007; Purpura et al., 2017). Moreover, EF is recognized as the strongest predictor of school readiness in the context of the development of pre-academic skills (Blair and Razza, 2007).

### Culture and Executive Function

Due to variations in social systems, beliefs, attitudes, and values across cultures, there has been an increased interest to explore the influence of culture on human developmental outcomes (Burrage et al., 2008; Suchodoletz et al., 2013; Wanless et al., 2013). Considering the importance of EF skills, the influence of culture on children's development of EF skills deserves attention (Ellefson et al., 2017). Findings from cross-cultural investigations so far suggest that, for instance, children from Eastern culture typically perform better on EF tasks than their Western counterparts (Sabbagh et al., 2006; Oh and Lewis, 2008; Lewis et al., 2009; Lan et al., 2011; Grabell et al., 2015). Different explanations have been proposed. For instance, Chen et al. (1998) mention early parental expectation of children' mastery of impulse control. While children from Eastern cultures (e.g., Chinese) are expected to have mastery of impulse control around 2 years of age, children from Western cultures are not expected the same until the preschool years (Chen et al., 1998). This could create opportunities for Chinese children to be taught the importance of self-control (Ellefson et al., 2017) and start practicing EF skills at an earlier age than their US counterparts (Sabbagh et al., 2006). There is also another explanation which is linked to the difference in parenting practices in the context of individualist vs. collectivist cultures (Schmitt et al., 2019). In a collectivist culture like China, children, for instance, are supposed to practice and learn how to behave in the society through adults modeling of the “acceptable” behaviors (Jian, 2009). On the other hand, individual choices and negotiation are valued elements in child rearing practices in individualistic cultures (Greenfield et al., 2006).

The other explanation comes from the difference in educational styles and schooling environments in the two cultural groups in general. According to Lan and colleages (2011), the difference of educational styles between countries (e.g., Chines and US) is the major reason for the variation in EF skills. While being able to control one's behavior, attention and concentration are a central element of Chinese education, self-expression and decision-making freedom are more in focus in US education (Song and Jinyu, 2016). On top of that, compared to the preschool settings in US, impulse control is exceedingly valued in Chinese preschools (Tobin et al., 1989). This might mean that the schooling environment in China is more supportive of the development of EF skills than the schooling environment in the US.

### Home Experience in Child Development

Home experience is proposed to be among the most important factors contributing to individual differences in early brain development. In this regard, there is a growing body of evidence demonstrating that children's home experiences such as family socioeconomic status (Mezzacappa, 2004; Noble et al., 2005; Sarsour et al., 2011), years of parental education (Ardila et al., 2005), parent-child interactions (Bernier et al., 2010, 2012; Rhoades et al., 2011; Blair et al., 2014; Linebarger et al., 2014; Lucassen et al., 2015; Meuwissen and Carlson, 2015), parental scaffolding/support (Hughes and Ensor, 2009; Bernier et al., 2010; Hammond et al., 2012; Hughes and Devine, 2017), social interactions (Moriguchi, 2014), and children's engagement in physical activity (Best, 2010) have all been implicated in the development of EF skills. Contrarily, children's experience of abuse and maltreatment (Belsky and de Haan, 2011; Pechtel and Pizzagalli, 2011), adverse parenting (Hughes and Devine, 2017), negative caregiving behaviors (Ardila et al., 2005), chaotic home environment (Hughes and Ensor, 2009; Brown et al., 2013; Vernon-feagans et al., 2016), and exposure to maternal depression (Hughes et al., 2013) have adverse effects on early development of EF.

### Play and Child Development

One of common home experiences of children that have valuable contribution to child learning and development is play (Ginsburg, 2007; Lin and Li, 2018). Beyond entertainment, play is important for cognitive, physical, social, and emotional development (Parmar et al., 2004; Tamis-LeMonda et al., 2004; Burdette and Whitaker, 2005; Johnson et al., 2005; Ginsburg, 2007; Göncü and Gaskins, 2011). Moreover, play gives children opportunities to develop their skills of working in groups and sharing, negotiating, and resolving conflicts with others (Pellegrini and Smith, 1998; Mcelwain and Volling, 2005).

Parental views about the importance of play for child development and the frequency at which children engage in different types and forms of play show substantial variation (Roopnarine, 2012). Roopnarine (2011) argued that the parents' views of the importance of play for child development fall along a continuum. Views that acknowledge the significant contribution of play in child development are at one end of the continuum. The other end of the continuum consists of views that considers play as something children do naturally just for amusement. The middle of the continuum represents views of parents that appreciate the specific benefits of play but favor academic activities for children.

Children's experience of play at home (see Farver and Wimbarti, 1995; Haight et al., 1997; Avornyo and Baker, 2018; Ihmeideh, 2019), and ability to gain the developmental benefit of play from playing with peers and adults are related to the ideas parents hold about play (Bulotsky-Shearer et al., 2016). Moreover, the beliefs parents hold about the importance of play for child development guide their decision-making processes to get involved in their children's play (Holmes, 2011). Parents who value play for its educational and cognitive benefits are more likely to encourage and assist their children's play through different means such us supplying numerous play materials to promote play opportunities, to join their children's play activities (e.g., parent-child play), and encourage and facilitate early peer contacts (see Parmar et al., 2004; Johnson et al., 2005). These opportunities in turn seem to create better developmental opportunities for children (Thorp et al., 1995; Carson and Parke, 1996; O'brien and Md-Yunus, 2007; Lin and Yawkey, 2014). Thus, investigating parental play beliefs, the level of involvement in their children's play and children's play experiences at home are important issues to be examined in relation to children's developmental outcomes (LaForett and Mendez, 2016).

### Culture and Play Beliefs

Even though child play is a universal activity, how it looks like is not necessarily the same across cultures (Farver, 1999; Gaskins, 1999). The description of play, the selection of play partners, the context under which play happens, and the time allocated to play are extensively affected by the cultural beliefs and experiences (Gaskins, 2006; Gaskins et al., 2006). Thus, there is no assurance to think that all communities hold similar views about the role of play in children's development and give equivalent play opportunities for their children (see Göncü et al., 2000; Parmar et al., 2004; Holmes, 2011; Roopnarine, 2011; Roopnarine and Jin, 2012; Roopnarine and Davidson, 2015). For instance, there are findings indicating that while adults with European cultural origin hold a strong play support belief that acknowledges the important role of play in child development (Haight et al., 1997; Parmar et al., 2004; Yahya and Wood, 2017), parents in some non-Western cultures or in low-income communities hold weak play support beliefs and appreciate the valuable contribution of academic training for children instead of play (Holmes, 2011; Yahya and Wood, 2017). In Mexican culture, in contrast to play activities, children's participation in work-related activities is thought to be far more important (Tamis-LeMonda et al., 2002). Similarly, the Caribbean teachers and parents emphasize the importance of academic activities for preschoolers instead of play activities (Leo-Rhynie et al., 2009; Logie, 2013). Likewise, Mayan mothers do not value the importance of play for their children's development (Rogoff et al., 1993). In their investigation, Farver et al. (1995) reported that Korean-American parents, compared to their Anglo-American counterparts, hold beliefs that academics is more valuable than play for their children, and they participate less in their children's pretend play. Children with parents holding less favorable attitudes toward play may be given less opportunities to play (Göncü et al., 2000).

On the whole, parental and cultural belief systems guide the play support beliefs parents hold and the types of play culture values (Roopnarine et al., 2003). The finding of a study examining cultural variations in the value, occurrence, frequency, and partners of play among toddlers/children, for instance, showed cultural disparities (Göncü et al., 2000). Mexican and Indonesian mothers who do not value the importance of play were found to give few chances to their children to play with them (Farver and Howes, 1993; Farver and Wimbarti, 1995). Besides, given the same form of play (e.g., parent-child play) in different cultures, the contents of play may demonstrate cross-cultural variations. For instance, the parent–child play activities in the Western technologically developed societies comprise of more language and gesture-oriented routines (Turkheimer et al., 1989) that may facilitate language and cognitive development in unique ways (Roopnarine, 2011). Thus, in order to fully comprehend the meaning of different play activities for children's development in different socio-cultural contexts, the cultural foundation of play should be taken into account (see Roopnarine, 2011).

### Socio-Cultural Contexts in Ethiopia and Hungary

#### Ethiopia

Ethiopia is a multi-ethnic and ecologically diverse East African country with a population of about 109 million (World Bank, 2019b) of which around eighty percent live in rural areas (World Bank, 2019a). The World Bank's 2018 report showed that Ethiopia's gross domestic product (GDP) was about 84.36 billion USD (World Bank, 2019b). In Ethiopia, while 71% of children (age 0–17) live with both biological parents, 17% of children live with either their biological mother or father only (Better Care Network, 2015). Most of the preschoolers are monolingual.

Kindergarten, which is mainly an urban phenomenon and left for the private sector, is a 3 year program for 4 to 6 year olds (Woldehanna, 2011). Accordingly, the government has very limited involvement in early childhood education such as providing some technical support and quality monitoring (Woldehanna, 2011). In order to improve the enrollment of young children in early childhood education in the country, “O” class and child-to-child programs, as part of early childhood education, have been launched very recently beyond the kindergarten program (MoE (Ministry of Education), 2016). Even though the Education and Training Policy (MoE, 1994) of the country underlines the importance of children's overall development during the preschool years, the preschools in the country run a very structured programs that focus more on academic activities and far less on play (see Tigistu, 2013; Admas, 2016). Interestingly, parents of preschoolers also hold academic focused beliefs that could drive preschools to focus on academic development instead of other aspects of development such as social-emotional development (Tigistu, 2013; Admas, 2016).

#### Hungary

Hungary is a country in East-Central Europe with a population of about 9.8 million of which 28.36% is reported as rural (World Bank, 2019c). According to World Bank's 2019 report, the country's GDP was 160.97 billion USD. Regarding ethnicity, the country is relatively homogenous (Józsa et al., 2018) and Hungarian is the official language. The majority of the Hungarian children are monolingual. It is estimated that about half a million children in Hungary grow up in single parent families (Coface Families Europe, 2018).

According to the Hungarian National Public Education law, children shall attend preschool education from the age of 3 until they reach compulsory school age (Act CXC of 2011 on National Public Education). As indicated in the Govn decree 363/2012 (XII. 17) on the Core Program for Kindergartens, the goal of preschool education in Hungary is “. promoting a harmonious physical and social development of the child's personality.” [363/2012 (XII. 17)]. The national core curriculum for kindergarten education regulates the pedagogical work in the kindergartens. The core program indicates the types of activities in the program as:

Play (as the fundament of preschool education); Rhyming and Storytelling; Drawing, Painting, Making Patterns, Handwork; Singing, Music, Singing Games, Children's Dances; Becoming Actively Acquainted with the Outer World (content in environmental and content in mathematics); Movement (physical activity); activities of a work nature (e.g., taking care of animals, plants); activity based learning (as cited in Böddi and Serfozo, 2019, p. 189).

### Present Study

Variations in social systems and values across cultures make the generalizability of the findings from child development studies across different populations and cultural groups difficult. Taking this point into account, many scholars have been investigating cross-cultural variations in human development including cognitive development. In this regard, findings have been indicating cultural consistencies and variabilities in child development. For instance, the cross-cultural comparison of children's performance on EF tasks show that children from Asian countries outperform their Western counterparts (e.g., Sabbagh et al., 2006; Lewis et al., 2009; Imada et al., 2013). However, such comparisons of EF skills have mostly been limited to children from Eastern and Western cultures. The present study, therefore, is believed to be one of the first efforts to enrich the literature on the areas by comparing samples from Ethiopia and Hungary.

Cross-cultural variations in children's home (such as parenting practices and parental expectation) and school experiences could take important weight in explaining the cross-cultural variations in children's EFs development (see Chen et al., 1998; Ellefson et al., 2017; Schmitt et al., 2019). However, very little is known about the importance of other home experiences of children such as play, sport and physical activities, arts and crafts activities, and fine motor activities, for example, in explaining the cross-cultural differences in children's EF skills. In line of this point in their investigation of the contribution of preschoolers' home experiences in the development of EF, Metaferia et al. (2020a) strongly recommended a follow-up study with a cross-cultural design taking the potential variations in preschoolers' home experience as a function of different socio-cultural contexts into account. We selected Ethiopia and Hungary for comparison taking their very different socio-cultural backgrounds into account, including the far reaching differences between the aims of and the parental expectations from early childhood education. Previous studies (Tigistu, 2013; Admas, 2016) suggested that children's academic development was very important for Ethiopian parents, even during the preschool years. As opposed to this, the focus of early childhood education in Hungary is on the physical and socio-emotional development of children (Govn decree 363/2012 XII. 17).

Therefore, the present investigation aimed at examining parents' beliefs about play and the purpose of preschool education, and preschoolers' home activities cross-culturally with preschoolers from Ethiopia and Hungary. The other purpose of the current investigation was to explore cross-cultural variations in the development of EF skills including inhibitory control, shifting and visual-spatial working memory. Finally, exploring cross-cultural variations in the links between preschoolers' home experiences and EF skills was another purpose of the study. To realize these purposes of the study, we formulated the research questions below:

- Do Ethiopian and Hungarian parents hold different views about the role of play in child development and the purpose of preschool education?
- Is there cross-cultural variation in the frequency of preschoolers' activities at home?
- Are there cross-cultural differences in preschoolers' EF skills including inhibition, switching, and visual-spatial working memory?
- Is there a cross-cultural difference in the relationships between the frequency of preschoolers' activities at home and their performance on the battery of EF tasks?

We hypothesized that there would be clear cross-cultural variations in parental attitudes about the purpose of preschool education between the two sample groups. More specifically, we speculated that Ethiopian parents' educational beliefs tend toward academic skills while Hungarian parents would tend to hold the belief that social-emotional development is the main purpose of preschool education. We also hypothesized that Hungarian parents would hold significantly stronger play support beliefs than their Ethiopian counterparts.

Based on previous studies (e.g., Metaferia et al., 2020a,b), we hypothesized that there might be cross-cultural variations in the relationship the frequency of children's home activities and their parents' play support would have with children's performance on the battery of EF task. To be more specific, it was found in a previous study that parental play support beliefs and preschoolers' frequency of breakfast at home were positively linked to children's inhibitory control skills in an Ethiopian sample (Metaferia et al., 2020b). On the other hand, in addition to parental play support beliefs, preschoolers' frequency of participation in pretend play was an important predictor of inhibitory control skills in a Hungarian sample (Metaferia et al., 2020a). We also speculated that children's frequency of participation in arts and crafts and in fine motor activities were important predictor of children's performance in VSWM task for Ethiopian and Hungarian samples, respectively. Regarding the second and third research questions, we made no hypothesis, thus, these parts of the study were exploratory.

## Materials and Methods

### Participants

The participants of the study were children attending preschools during the time of data collection and their parents. Preschoolers with special needs (recognized by the preschools), and multilinguals were excluded from the study. Preschools from which the participant children were selected represent lower to middle-class areas in their respective countries. The education level of parents and family household annual income are presented in Table 1.

**Table 1:** Samples demographics.

	**Ethiopian sample**		**Hungarian sample**	
**Variables**	**Frequency (Mother/father)**	**Percent (Mother/father)**	**Frequency (Mother/father)**	**Percent (Mother/father)**
**Parents' education level**				
Elementary school complete	13/9	9/7	6/8	5/6
High school complete	39/34	28/25	37/50	29/39
College diploma	43/27	31/19	37/26	29/20
University degree	33/38	24/27	38/31	30/24
Graduate degree (master's or above)	9/30	7/22	8/12	6/9
Not reported	2/1	1/1	1/-	1/-
**Gross household annual income (in Birr)**				
0–24,999	21	15		
25,000–49,999	20	14		
50,000–79,999	26	19		
80,000–99,999	17	12		
100,000–149,999	22	16		
150,000–199,999	11	8		
200,000 or more	19	14		
Not reported	3	2		
**Gross household annual income (in Forint)**				
0–49.999			–	–
50.000–99.999			4	3
100.000–199.999			7	6
200.000–299.999			15	12
300.000–399.999			20	16
400.000–499.999			23	18
500.000–599.999			19	15
600.000–699.999			13	10
700.000 or above			19	15
Not reported			7	6

#### Ethiopia

One hundred and thirty-nine preschoolers (62 boys, and 77 girls; *M*_age_ = 63.83 months; *SD* = 7.68 months; age range = 44–84 months) and their parents (63 male and 76 females; *M*_age_ = 36.66 years; *SD* = 7.14 years; age range = 20–60 years) recruited from nine preschools in the capital, Addis Ababa, participated in the study. All participating preschoolers were Amharic monolinguals. Family members of the participant preschoolers who supplied the requested information for the study were preschoolers' father (43.2%), mother (53.2%), grandfather (0.7%), and grandmother (1.4%).

#### Hungary

One hundred and twenty-seven preschoolers (61 boys, 66 girls; *M*_age_ = 62.06 months; *SD* = 9.37 months; age range = 45–84 months) and their parents (17 male and 110 females; *M*_age_ =37.71 years; *SD* = 5.97 years; age range = 22–63 years) from seven preschools in the capital, Budapest, took part in the study. Seventy-six of the total Hungarian participants in the current study also participated in a previous study (Metaferia et al., 2020a) of ours. All participating preschoolers were Hungarian monolinguals. Preschoolers' caregivers who provided the required information about the participant child were their father (7.9%), mother (90.6%), grandfather (0.8%), and grandmother (0.8%).

Independent sample *t*-tests depicted that there were no significant differences between the two samples on children's age [(*M*_Ethiopia_ = 63.83, *SD*_Ethiopia_ = 7.68; M_Hungary_ = 62.06, *SD*_Hungary_ = 9.37); *t* (241.98) = 1.67, *p* = 0.097] or average parental education level [(*M*_Ethiopia_ = 0.3.13, *SD*_Ethiopia_ = 1.05; *M*_Hungary_ = 2.98, *SD*_Hungary_ = 0.95); *t* (260) = 1.17, *p* = 0.245].

### Procedure

Preschools from Addis Ababa and Budapest were contacted to recruit participants for the present study. After obtaining written consent of preschool directors, information sheet and consent paper were sent to parents of preschoolers to request for their participation including their preschoolers in the present study. When parents agreed to participate by signing the consent form, the questionnaires were sent to them. When parents returned the filled in questionnaire to preschools, neuropsychological tests measuring EF skills were administered to their children by trained research assistants. The tests were administered individually at a separate quiet room in their respective preschools. The sequence of the tasks was kept the same for all participants: the go/no-go task, the switching task, and the visual-spatial working memory task. The test session lasted about 20–25 min for each child. The procedure applied in the present investigation was permitted by the Research Ethics Committee of the Faculty of Education and Psychology at ELTE Eötvös Loránd University's (issue number: 2017/209).

### Measures

A questionnaire with four parts (demographic information, parental views on the goal of preschool education, parental play beliefs, and the child's home activities) was used in order to collect data from parents. Originally, the questionnaire was prepared in English and translated into the target languages (Amharic and Hungarian). As an average, the questionnaire took 30 min to be filled in.

#### Demographic Information

The demographic information consisted of the child's gender, age, year of enrolment in preschool, and relationship with the respondent, parents' highest level of education, and the family size and gross annual income was collected. A five-point scale ranging from elementary school completed (1) to graduate degree/Master's or above (5) was applied to measure the educational level of both parents. A seven- and a nine-point categorical scales were applied in measuring the family's gross annual income for Ethiopian and Hungarian samples, respectively. The z-score of the average education of parents and the z-score of annual income per family size were averaged to create SES variable.

#### Parental Play Beliefs Scale

Parental play beliefs were measured using the Parental Play Beliefs Scale (PPBS) developed by Fogle and Mendez with sample of low-income mothers with children enrolled in Head Start program (Fogle and Mendez, 2006). The PPBS consists of 25 items with a five-point Likert-scale ranging from 1 (disagree) to 5 (very much agree). In the scale validation study, a principal component analysis yielded two subscales, namely Play Support and Academic Focused beliefs (Fogle and Mendez, 2006). The Play Support scale includes 17 items (e.g., “I can help my child learn to control his or her emotions during play,” and “through play my child develops new skills and abilities”) and the Academic Focused scale consists of 8 items (e.g., “I would rather read to my child than play together,” and “I do not think my child learns important skills by playing”).

In the present investigation, item differential function (DIF) analysis was carried out on the scale using ordinal logistic regression technique (see Zumbo, 1999) in order to examine whether the items function similarly in the two sample groups. Accordingly, an item (item 2) from Academic Focused and four items (item 21, 22, 23, and 24) from Play Support scales were excluded from the scale as they functioned differently in the two sample groups. In the scale validation study, the subscales depicted internal consistency of α = 0.90 and 0.73 for Play Support and Academic Focused scales, respectively (Fogle and Mendez, 2006). The subscales also showed a good level of internal consistency in the current study (Ethiopian sample: Play Support = 0.89, Academic Focused = 0.70; Hungarian sample: Play Support = 0.80, and Academic Focused = 0.63) after removing the items functioning differently. The bivariate correlation between the two subscales depicted similar sized, significant negative correlations in both samples (*r*_Ethiopia_ = −0.41, *p* < 0.001, and *r*_Hungary_ = −0.39, *p* < 0.001).

#### Parents' Beliefs About the Purpose of Preschool Education Scale

A ranking-order scale with 7 items, developed by Metaferia et al. (2020a), was used to measure parents' views on the purpose of preschool education. The scale consists of a list of potential purposes of preschool education (general knowledge, cognitive skills, language development, social skills, enjoy themselves, emotional well-being, and academic skills). Parents are asked to rank the purposes of preschool education listed based on their importance (1 as the most important to 7 as the least important) based on their personal beliefs. Average rankings for each item were calculated in the two samples.

#### Child's Home Activities Scale

The Child's Home Activities Scale (CHAS), developed by Metaferia et al. (2020b), was used to measure preschoolers' frequency of participation in the selected home activities. The activities included in the scale were planned as individual indicators of preschoolers' activities at their home. The scale was designed to gather information about different aspects of children's everyday lives such as frequency of engagement in different type/form of play (e.g., pretend play, motor play, solitary play, and peer play), academic-related activities, art and craft activities and sports and physical activities. Furthermore, frequency of having breakfast at home, and spending mealtime together with family were addressed in the scale intending to gather information about whether or not children get nutrition before preschool as opposed to “mealtime together” which was designed to examine its social implications. The scale consists of ten Likert-scaled items ranging from “very rarely /less than once a week” (1) to “very frequently /most of the time during the day” (5). In the scale, parents were requested to rate the frequency at which their children participate in the home activities included in the scale: breakfast at home, spending meal time with family, academic-related activities, pretend play, motor play, fine motor activities, arts and crafts, solitary play, peer play, and sports and physical activity.

#### Translation of the Survey

The English version of the questionnaire was carefully translated to Amharic and Hungarian. Three independent translators of which two were educational psychologists with a master's degree and one applied developmental psychologist with a PhD level of education were involved in the translation of the Amharic version of the questionnaire (see Metaferia et al., 2020b). The translators were native speakers of Amharic and fluent in English. Upon completion, the three translations were collected and checked for the discrepancies by the first author and made important amendments. The translation of the Hungarian version of the questionnaire involved two independent native speakers of Hungarian and fluent in English. The collected translations from the translators were cross checked for their discrepancies and made important revisions by the second and third authors (see Metaferia et al., 2020a).

#### Executive Functions Measures

Three computerized neuropsychological tests, a go/no-go task, a switching task and a Mr. Peanut task, were administered to children to measure their inhibitory control, switching, and visual-spatial working memory, respectively. The first two tests were run using 1.85.1 version of PsychoPy (Psychological Software Tools; Peirce, 2008).

#### Go/No-Go Task

A fish and shark go/no-go task was applied to measure the inhibitory control skills of the participant preschoolers (see Wiebe et al., 2012). The task consisted of a practice and a test session. The practice session consisted of 4-go and 2-no-go trials demanding children to respond to the target and withhold for the non-target stimuli. The testing session consisted of 40-go and 20-no-go trials where a stimulus appears on a laptop screen for 1,500 ms unless a child responded earlier. Children were expected to respond to the target (i.e., when the target/fish stimulus appears on the screen) as quickly as possible by pressing the space bar in a laptop keyboard and withhold for the non-target (i.e., when the non-target/shark stimulus appears on the screen). Using frequency of correctly responding to go trials and no-go trials, sensitivity (*d'*) was calculated for each child following the signal detection theory (see Macmillan and Creelman, 2005).

#### Switching Task

A cat and tiger go/no-go task with four blocks was used to measure shifting skill of the participant children (Metaferia et al., 2020a,b). The go and no-go stimuli were counterbalanced by switching the target and non-target stimuli as the task moves from one block to the next block. Correspondingly, participants were informed the target and non-target stimuli before the beginning of every block. Each block consisted of 16 go and 8 no-go trials where each stimulus appeared on the screen for 1,200 ms if the child did not respond to it earlier. Before the beginning of the actual test, a practice session involving 4-go and 2-no-go trials, was carried out. In order to estimate the switching task score of each child, sensitivity (*d'*) was calculated for each block based on signal detection theory. Based on the sensitivity scores of each child for every block, the sensitivity score differences between switching blocks was calculated (i.e., sensitivity of block 3 minus block 1 and sensitivity of block 4 minus block 2). Next, the average of the two differences was calculated as switching score of every child (see Metaferia et al., 2020b).

#### Mr. Peanut Visual Spatial Working Memory Task

The children's visual spatial working memory (VSWM) was measured using Mr. Peanut visual special working memory task designed based on work of Morra (1994). Mr. Peanut is a “crown-like” picture that appears on a computer screen with stickers on his body and disappears for a while and reappears on the screen again without stickers. The stickers can appear 14 different possible locations on Mr. Peanut body part. The task for the children is to put sticker on Mr. Peanut's body using their memory after he removed them. The task begins with a practice session consists of 3 trials where children learn how to put stickers on Mr. Peanut's body by memorizing their locations before they are removed. The task progresses step-by-step starting from one sticker to seven stickers at a time where in every stage three attempts were given. In order to move to the next level of the task the child should at least correctly respond to one of the three trials at the present stage of the task. At each level of the task, correctly responding to one or at least two of the three trials results scores of 0.33 and 1, respectively. The total score was calculated by adding an individual score the child earned at each of the stages he/she gone through.

### Data Analyses

SPSS v20.0 for Windows (IBM Corporation, Armonk, NY, USA) was used in conducting the statistical analyses. All variables used in the analyses were found to be normally distributed. Independent samples *t*-tests were applied to compare preschoolers' activities at home and their performance in EF tasks, and parental play beliefs between the two samples. Bivariate correlation analyses were used to examine the relationship preschoolers' EFs could have with parental play beliefs and preschoolers' home activities. We also built two-step hierarchical regression models explaining preschoolers' EF skills using parental play beliefs and preschoolers' home activities as predictors for each sample separately. Accordingly, children's age and SES were entered in the first step as control variables. Next, the significant correlates of the corresponding EF component from home activity and parental play belief variables were entered together into the regression equations. Moreover, Fisher z-transformation was carried out to make comparison of the correlation between preschoolers' home experiences (preschoolers' home activity and their parents' play beliefs) and their performance on the battery of EF tasks between the two sample groups. Furthermore, Friedman-tests were used to analyze parents' priorities regarding the purpose of preschool education in the two groups separately. *Post-hoc* test using a Wilcoxon signed-rank tests were also carried out to examine the significance of the differences in their beliefs in the two samples independently.

## Results

Table 2 presents descriptive statistics for EF and home experience measures in the two samples.

**Table 2:** Descriptive statistics for EF and home experience measures in two samples.

	**Ethiopian sample**				**Hungarian sample**			
**Variables**	***N***	**Mean**	**SD**	**Min–Max**	***N***	**Mean**	**SD**	**Min–Max**
SES	132	0.00	0.91	−1.68 to 2.17	113	0.01	0.82	−2.13 to 1.65
**Child's EF**								
Inhibitory control	134	2.73	0.85	0.00 to 4.20	124	2.92	0.90	0.00 to 4.20
Cognitive flexibility	124	−0.10	0.64	−1.51 to 1.36	121	−0.18	0.67	−2.16 to 1.53
VSWM	138	2.62	0.77	0.67 to 4.33	118	2.54	0.92	0.33 to 4.33
**Child's home activity**								
Academic skills practice after preschool	135	3.35	1.20	1 to 5	120	2.72	1.40	1 to 5
Mealtime together with family	137	3.77	1.02	1 to 5	127	4.02	1.10	1 to 5
Breakfast at home	137	3.56	1.01	1 to 5	125	3.72	0.93	2 to 5
Engage in pretend play	138	3.37	1.05	2 to 5	127	3.48	1.31	1 to 5
Engage in motor play	137	3.93	1.22	1 to 5	125	4.12	1.10	1 to 5
Engage in fine-motor activities	139	3.35	1.24	1 to 5	127	3.68	1.09	1 to 5
Participate in arts and crafts	135	3.08	1.22	1 to 5	127	2.52	1.13	1 to 5
Engage in solitary play	136	2.85	1.51	1 to 5	126	3.75	1.14	1 to 5
Play with peers	136	3.80	1.08	1 to 5	126	3.74	1.29	1 to 5
Do sports and physical activities	136	2.75	1.43	1 to 5	127	3.41	1.03	1 to 5
**Parents' play beliefs**								
Parental belief: Academic focused	123	16.35	5.27	6.00 to 30.00	119	10.52	2.70	6.00 to 18.00
Parental belief: Play support	131	55.38	8.75	31.00 to 65.00	123	56.43	4.95	45.00 to 65.00

*Research Question 1:* Do Ethiopian and Hungarian parents hold different views about the role of play in child development and the purpose of preschool education?

As can be seen in Table 3, the independent-samples *t*-test showed that there was no significant difference between the play support beliefs that the Ethiopian and the Hungarian parents held. However, Ethiopian parents held significantly stronger academic focused beliefs than their Hungarian counterparts.

**Table 3:** *T*-test statistics for home experience measures between the two samples.

	**Ethiopian sample**		**Hungarian sample**		
**Home experience Variables**	**Mean**	**SD**	**Mean**	**SD**	***t(df)***
**Child's home activity**					
Academic skills practice after preschool	3.36	1.20	2.72	1.40	3.94 (236.14)^**^
Mealtime together with family	3.77	1.02	4.02	1.10	*−1.92 (262)*
Breakfast at home	3.56	1.01	3.72	0.93	*−1.32 (260)*
Engage in pretend play	3.37	1.05	3.48	1.31	*−0.75 (241.45)*
Engage in motor play	3.93	1.22	4.12	1.10	*−1.34 (260)*
Engage in fine-motor activities	3.35	1.24	3.68	1.09	−2.27 (263.64)^*^
Participate in arts and crafts	3.08	1.22	2.52	1.13	3.87 (260)^**^
Engage in solitary play	2.85	1.51	3.75	1.14	−5.42 (249.66)^**^
Play with peers	3.80	1.08	3.74	1.29	*0.430 (245.07)*
Do sports and physical activities	2.75	1.43	3.41	1.03	−4.32 (245.21)^**^
**Parents' play beliefs**					
Parental belief: Academic focused	16.35	5.27	10.52	2.70	10.89 (183.42)^**^
Parental belief: Play support	55.38	8.75	56.43	4.95	*−1.19 (207.96)*

* *p < 0.05*;

** *p < 0.001*.

A Friedman ANOVA and Wilcoxon signed-rank tests were carried out to assess priorities in parental educational beliefs in both samples, separately. Table 4 presents the summary of the median and interquartile range for parental educational beliefs in the two samples. The result showed that parents hold different prioirities for the different educational goals in both samples (Ethiopia: χ2(6) = 83.93, *p* < 0.001; and Hungary: χ2(6) = 253.43, *p* < 0.001). *Post-hoc* test using a Wilcoxon signed-rank tests with Bonferroni-adjusted significance level of *p* < 0.0024, was carried out. The summary of the Wilcoxon signed-rank test using the z-statistics and significance values are presented in Table 5. As Table 4 shows that while Ethiopian parents held an educational belief that prioritizes academic and cognitive development, Hungarian parents held the educational beliefs that prioritize social-emotional development and enjoyment for their children.

**Table 4:** The median and interquartile range of parental beliefs about the purpose of preschool education for Ethiopian and Hungarian samples.

	**Ethiopian sample**		**Hungarian sample**	
**Purpose of preschool education**	**Median**	**Interquartile range**	**Median**	**Interquartile range**
General knowledge	4.00	2.00–7.00	5.00	4.00–6.00
Cognitive skills	2.00	1.00–4.00	4.00	3.00–5.00
Language development	4.00	2.00–5.00	5.00	4.00–6.00
Social skills	3.00	2.00–5.00	2.00	1.00–3.00
Enjoy themselves	4.00	2.00–6.00	2.00	2.00–4.00
Emotional well-being	5.00	3.00–6.20	2.00	1.00–3.75
Academic skills	3.00	1.00–5.00	7.00	5.00–7.00

**Table 5:** Summary of Wilcoxon signed-ranks test using the *Z* statistic and significance value.

**Purpose of preschool education**	**Ethiopian sample**		**Hungarian sample**	
	**Z**	***p***	**Z**	***p***
General knowledge vs. cognitive development	−6.42	0.000^*^	−2.84	0.005
General knowledge vs. language skill development	−3.73	0.000^*^	−0.41	0.680
General knowledge vs. social skill development	−2.95	0.003	−7.41	0.000^*^
General knowledge vs. enjoyment	−1.04	0.297	−6.29	0.000^*^
General knowledge vs. emotional well-being	−1.15	0.25	−6.83	0.000^*^
General knowledge vs. academic skill development	−4.16	0.000^*^	−4.16	0.000^*^
Cognitive development vs. language skill development	−3.82	0.000^*^	−2.28	0.023
Cognitive development vs. social skills development	−3.82	0.000^*^	−6.72	0.000^*^
Cognitive development vs. enjoyment	−4.60	0.000^*^	−4.78	0.000^*^
Cognitive development vs. emotional well-being	−6.85	0.000^*^	−6.42	0.000^*^
Cognitive development vs. academic skills development	−2.47	0.013	−6.00	0.000^*^
Language skills development vs. social skills development	−0.37	0.715	−7.94	0.000^*^
Language skills development vs. enjoyment	−2.14	0.032	−5.86	0.000^*^
Language skills development vs. emotional well-being	−4.72	0.000^*^	−6.97	0.000^*^
Language skills development vs. academic skills development	−0.78	0.436	−4.21	0.000^*^
Social skills development vs. enjoyment	−1.99	0.047	−1.71	0.087
Social skills development vs. emotional well-being	−4.66	0.000^*^	−0.90	0.370
Social sills development vs. academic skills development	−0.95	0.340	−8.68	0.000^*^
Enjoyment vs. emotional well-being	−3.14	0.002^*^	−1.10	0.271
Enjoyment vs. academics skills development	−2.86	0.004	−7.78	0.000^*^
Emotional well-being vs. academics skills development	−4.68	0.000^*^	−8.09	0.000^*^

* *p < 0.0024 (Bonferroni correction)*.

*Research Question 2:* Is there cross-cultural variation in the frequency of preschoolers' activities at home?

As shown in Table 3, the result of independent samples *t*-tests showed that Ethiopian preschoolers practiced academic skills and participated in arts and crafts activities after preschool significantly more often than their Hungarian peers. On the other hand, Hungarian preschoolers engage in fine-motor activities, solitary play, and sports and other physical activities significantly more often than their Ethiopian counterparts. However, there were no significant differences regarding frequency of mealtime together with family, breakfast at home, pretend play, motor play or peer play.

*Research Question 3:* Are there cross-cultural differences in preschoolers' EF skills including inhibition, switching, and visual-spatial working memory?

Independent-samples *t*-tests were carried out in order to examine if there were cross-cultural variations in children's performance on the battery of EF tasks between the two samples. The results indicated that there were no statistically significant difference in inhibitory control [(*M*_Ethiopia_ = 2.73, *SD*_Ethiopia_ = 0.85; *M*_Hungary_ = 2.92, *SD*_Hungary_ = 0.90); *t* (251.61) = −1.70, *p* = 0.091], switching [(*M*_Ethiopia_ = −0.10, *SD*_Ethiopia_ = 0.64; *M*_Hungary_ = −0.18, *SD*_Hungary_ = 0.67); *t* (241.86) = 0.94, *p* = 0.348] or VSWM [(*M*_Ethiopia_ = 2.62, *SD*_Ethiopia_ = 0.77; *M*_Hungary_ = 2.54, *SD*_Hungary_ = 0.92); *t* (228.00) = 0.78, *p* = 0.435] between the two samples.

*Research Question 4:* Is there cross-cultural variation in the relationships between the frequency of preschoolers' activities at home and their performance on the battery of EF tasks?

As shown in Table 6, the bivariate correlation analyses indicated that the sociodemographic variables (age of preschoolers and SES) showed significant, positive correlations with preschoolers' performance in all the three EF tasks in both countries. Frequency of participation in pretend play, fine motor activities and arts and crafts activities showed significant positive correlation with preschoolers' performance on Mr. Peanut VSWM task in both countries. On the other hand, frequency of breakfast at home and peer play were also significant correlates of children's score on VSWM task in the Ethiopian sample. Frequency of breakfast at home, pretend play, peer play and parental play support were significant correlates of inhibitory control in both samples. On the other hand, while preschoolers' frequency of participation in motor play showed significant, positive association with inhibitory control in the Hungarian sample, frequency of mealtime together with family, participation in fine motor activities, and in sports and physical activities showed significant, positive correlations with inhibitory control in Ethiopia. We also found that parental play support and preschoolers' frequency of pretend play and peer play displayed significant, positive correlations with preschoolers' performance in the switching task in both countries. Additionally, frequency of breakfast at home and participation in sport and physical activities showed significant, positive correlations with shifting skills in Ethiopia and Hungary, respectively.

**Table 6:** Bivariate correlations among demographics, home activities, parental play beliefs, and executive functions variables for Ethiopian and Hungarian samples.

	**1**	**2**	**3**	**4**	**5**	**6**	**7**	**8**	**9**	**10**	**11**	**12**	**13**	**14**	**15**	**16**	**17**
Child's Age	1	0.18 *N =* 112	0.36^**^N = 117	0.35^**^ *N =* 123	21^*^N = 120	0.23^*^ *N =* 119	0.02 *N =* 126	0.11 *N =* 124	0.09 *N =* 126	0.25^*^ *N =* 124	0.00 *N =* 126	0.08 *N =* 126	0.01 *N =* 125	0.06 *N =* 125	0.07 *N =* 126	0.00 *N =* 118	0.13 *N =* 122
SES	0.07 *N =* 126	1	0.24^*^N = 104	0.37^**^ *N =* 110	25^**^N = 107	−0.09 *N =* 107	0.09 *N =* 113	0.16 *N =* 111	0.20^*^N = 113	0.00 *N =* 112	0.00 *N =* 113	−0.02 *N =* 113	0.09 *N =* 112	0.17 *N =* 112	0.07 *N =* 113	−0.20^*^ *N =* 108	0.31^**^N = 110
VSWM	0.36^**^N = 132	0.35^**^ *N =* 131	1	0.31^**^ *N =* 117	0.40^**^N = 112	0.13 *N =* 112	−0.02 *N =* 118	0.01 *N =* 116	0.19^*^N = 118	0.09 *N =* 116	0.23^*^N = 118	0.19^*^ *N =* 118	0.03 *N =* 117	0.13 *N =* 115	0.11 *N =* 118	0.08 *N =* 111	0.09 *N =* 114
Inhibitory control	0.23^**^N = 128	0.45^**^ *N =* 127	0.44^**^N = 133	1	0.35^**^N = 118	0.03 *N =* 117	0.05 *N =* 124	0.20^*^ *N =* 122	0.33^**^N = 124	0.18^*^ *N =* 122	0.11 *N =* 126	0.07 *N =* 124	0.06 *N =* 123	0.21^*^ *N =* 123	0.16 *N =* 124	0.02 *N =* 116	0.40^**^N = 120
Cognitive flexibility	22^*^N = 118	22^*^ *N =* 118	32^**^N = 118	20^*^ *N =* 119	1	−0.11 *N =* 115	0.17 *N =* 121	0.10 *N =* 119	0.19^*^N = 121	0.06 *N =* 119	0.14 *N =* 121	−0.02 *N =* 121	0.00 *N =* 120	0.22^*^ *N =* 120	0.19^*^N = 121	−0.11 *N =* 113	0.23^*^N = 118
Academic–related activities	0.21^*^N = 130	−0.04 *N =* 128	0.06 *N =* 134	−0.10 *N =* 130	−0.04 *N =* 120	1	0.15 *N =* 120	−0.02 *N =* 119	−0.03 *N =* 120	0.01 *N =* 119	−0.01 *N =* 120	0.11 *N =* 120	−0.10 *N =* 119	−0.06 *N =* 118	−0.07 *N =* 120	0.44^**^ *N =* 116	−0.11 *N =* 117
Mealtime with family	0.04 *N =* 131	0.15 *N =* 130	0.12 *N =* 136	0.29^**^ *N =* 132	0.04 *N =* 122	0.12 *N =* 133	1	0.26^**^ *N =* 125	0.05 *N =* 127	0.24^**^ *N =* 125	−0.10 *N =* 127	−0.09 *N =* 127	0.12 *N =* 126	0.22^*^ *N =* 126	0.14 *N =* 127	0.15 *N =* 119	−0.11 *N =* 123
Breakfast at home	0.23^**^N = 131	0.30^**^ *N =* 130	0.24^**^N = 136	0.42^**^ *N =* 132	0.27^**^N = 122	0.05 *N =* 133	0.03 *N =* 136	1	0.07 *N =* 125	0.04 *N =* 123	−0.01 *N =* 125	−0.02 *N =* 125	−0.08 *N =* 124	0.22 *N =* 124	0.14 *N =* 125	−0.03 *N =* 118	0.03 *N =* 121
Pretend play	0.16 *N =* 133	0.25^**^ *N =* 131	0.25^**^N = 137	0.41^**^ *N =* 133	0.20^*^N = 123	0.07 *N =* 134	0.31^**^N = 136	0.26^**^ *N =* 136	1	0.19^*^ *N =* 125	0.20^*^N = 127	0.09 *N =* 127	0.29^**^N = 126	0.11 *N =* 126	0.10 *N =* 127	−0.23^*^ *N =* 119	0.19^*^N = 123
Motor play	0.22^*^N = 131	0.02 *N =* 130	0.02 *N =* 136	0.16 *N =* 132	0.12 *N =* 122	−0.02 *N =* 133	0.25^**^N = 136	0.10 *N =* 137	0.33^**^N = 136	1	0.04 *N =* 125	0.04 *N =* 125	0.18 *N =* 124	0.10 *N =* 122	0.12 *N =* 125	−0.06 *N =* 118	0.13 *N =* 122
Fine motor activities	0.07 *N =* 133	0.05 *N =* 132	0.19^*^N = 138	0.17^*^ *N =* 134	0.16 *N =* 124	−0.08 *N =* 135	0.37^**^N = 137	0.09 *N =* 137	0.30^**^N = 138	0.36^**^ *N =* 137	1	0.45^**^ *N =* 127	0.04 *N =* 126	0.09 *N =* 126	−0.04 *N =* 127	−0.10 *N =* 119	0.22^*^N = 123
Arts and crafts	0.00 *N =* 129	0.12 *N =* 128	0.19^*^N = 134	0.04 *N =* 130	0.02 *N =* 120	0.10 *N =* 131	0.01 *N =* 135	0.01 *N =* 134	−0.03 *N =* 134	0.15 *N =* 134	0.24^**^N = 135	1	0.05 *N =* 126	−0.07 *N =* 126	−0.10 *N =* 127	0.04 *N =* 119	0.02 *N =* 123
Solitary play	0.07 *N =* 132	−0.03 *N =* 129	0.06 *N =* 135	0.16 *N =* 131	−0.01 *N =* 121	−0.14 *N =* 132	0.18^*^N = 134	0.03 *N =* 134	0.21^*^N = 135	0.17^*^ *N =* 133	0.13 *N =* 136	0.02 *N =* 132	1	−0.01 *N =* 125	−0.02 *N =* 126	−0.15 *N =* 118	0.03 *N =* 122
Peer play	0.06 *N =* 130	0.03 *N =* 129	0.19^*^N = 135	0.30^**^ *N =* 132	0.18^*^N = 121	−0.04 *N =* 132	0.15 *N =* 134	0.14 *N =* 134	0.23^**^N = 135	0.33^**^ *N =* 134	0.10 *N =* 136	0.10 *N =* 132	0.05 *N =* 133	1	0.37^**^N = 126	−0.09 *N =* 119	0.02 *N =* 123
Sports and physical activities	0.19^*^N = 130	0.08 *N =* 129	0.01 *N =* 135	0.25^**^ *N =* 131	0.09 *N =* 121	−0.11 *N =* 132	19^*^N = 134	−0.02 *N =* 135	0.16 *N =* 135	0.41^**^ *N =* 135	0.06 *N =* 136	0.11 *N =* 132	0.16 *N =* 133	0.19^*^ *N =* 133	1	−0.09 *N =* 119	0.00 *N =* 123
PB: Academic focused	−0.10 *N =* 118	−0.27^**^ *N =* 116	−0.18 *N =* 123	−0.27^**^ *N =* 119	−0.19 *N =* 108	0.33^**^ *N =* 119	−0.11 *N =* 122	−0.07 *N =* 123	−0.04 *N =* 122	−0.10 *N =* 123	0.00 *N =* 123	0.13 *N =* 121	−0.18^*^N = 121	00 *N =* 120	−0.13 *N =* 121	1	−0.39^**^N = 116
PB: Play support	0.10 *N =* 125	0.20^*^ *N =* 124	0.10 *N =* 130	0.54^**^ *N =* 126	0.23^*^N = 117	−0.09 *N =* 127	0.31^**^N = 129	0.27^*^ *N =* 129	0.44^**^N = 130	0.43^**^ *N =* 129	0.32^**^N = 131	0.02 *N =* 128	0.34^**^N = 128	0.33^**^ *N =* 128	0.33^**^N = 128	−0.41^**^ *N =* 118	1

*PB, Parental belief; Correlations for the Ethiopian sample in the bottom diagonal; correlations for the Hungarian sample in the top diagonal;*

* *p < 0.05*;

** *p < 0.001*.

Comparisons of significant correlations of preschoolers' home experiences and their performance on the battery of EF tasks between the two sample groups were carried out using Fisher z-transformation (single sided test). The correlations between frequency of pretend play and inhibitory control, peer play and inhibitory control, and parental play support and inhibitory control were comparable in the two countries (zs < .1.40, ns). However, correlation between breakfast and inhibitory control were significantly higher in case of the Ethiopian sample than their Hungarian counterparts (z = 1.93, *p* = 0.027). The correlation between frequency of pretend play and VSWM, frequency of fine motor and VSWM, and frequency of arts and crafts and VSWM were also comparable in the two samples (zs <0.50, ns). Moreover, the correlations between pretend play and switching, peer play and switching, and parental play support and switching were also equivalent (zs < 0.32, ns) (See the Supplementary Material).

Next, the contribution of parental play beliefs and preschoolers' home activities to the EFs were examined in separate regression models for each country. Based on the results of the correlational analyses, all the variables that had significant correlations with the corresponding component of EF were entered simultaneously into a regression model, after controlling for preschoolers' age and SES.

In Ethiopia, preschoolers' age and SES together accounted for a significant proportion of the variance (24%) in the inhibitory control [*F*_(2, 108)_ = 16.56, *p* < 0.001]. Next, frequency of mealtime together with family, breakfast at home, pretend play, fine motor activities, peer play, sport and physical activities, and parental play support were entered into the model predicting inhibitory control. Only frequency of breakfast at home, pretend play, and parental play support were found significant predictors in the model that accounted for an additional 20% variance in inhibitory control. After removing non-significant predictors that included mealtime, fine motor activities, peer play, and sport and physical activities from the equation, the model was significant [*F*_(5, 105)_ = 15.97, *p* < 0.001] and explained 43% of the variance in preschoolers' performance in inhibitory control task. In the model, while the parental play support variable was a medium-sized predictor (β = 0.32, *p* < 0.001), frequency of breakfast (β = 0.17, *p* = 0.035) and pretend play (β = 0.17, *p* = 0.041) variables were small-sized predictors.

Another regression equation was built for the Ethiopian sample to examine the contribution of preschoolers' home activities to their VSWM task performance, after putting age and SES in control. Preschoolers' age and the SES variables together accounted 21% of the variance in VSWM [*F*_(2, 118)_ = 15.98, *p* < 0.001]. Based on the results of correlational analyses, frequency of breakfast at home, pretend play, fine motor activities, arts and crafts activities, and peer play were simultaneously entered into the equation predicting VSWM. The result showed that only the frequency of participation in arts and crafts activities was a significant predictor next to sociodemographic variables that improved the model by 3%. After removing the variables not significantly contributing (breakfast at home, pretend play, fine motor activities, and peer play) to the model, the final model explained 24% [*F*_(3, 117)_ = 12.42, *p* < 0.001] of the variance in VSWM. Finally, in order to examine the contribution of preschoolers' home experience in their performance on switching task, another regression model was built. Preschoolers' age and SES were included to the equation as the first step. However, the model was not significant [*F*_(2, 97)_ = 2.61, *p* = 0.079]. Table 7 depicts the summary of the final model.

**Table 7:** Summary of hierarchical multiple regression models predicting executive function skills in Ethiopian sample.

	**Inhibitory control**				**Visual–spatial working memory**				**Switching**			
	**Step 1**		**Step 2**		**Step 1**		**Step 2**		**Step 1**		**Step 2**	
	β	*95% CI*	β	*95% CI*	β	*95% CI*	β	*95% CI*	β	*95% CI*	β	*95% CI*
Age	0.22	(0.006,0.042)^**^	0.15	(0.000,0.033)^*^	0.32	(0.015,0.047)^***^	0.32	(0.016,0.048)^***^	0.09	(−0.009,0.025)	0.03	(−0.015,0.020)
SES	0.41	(0.230,0.545)^***^	0.24	(0.081,0.378)^**^	0.31	(0.127,0.398)^***^	0.29	(0.111,0.380)^***^	0.20	(0.004,0.284)^*^	0.12	(−0.068,0.231)
Breakfast			0.17	(0.010,0.282)^*^							0.18	(−0.029,0.249)
Pretend play			0.17	(0.005,0.258)^*^							0.04	(−0.112,0.160)
Play support			0.32	(0.016,0.049)^***^							0.12	(−0.007,0.025)
Arts and crafts							0.17	(0.006,0.204)^*^				
Peer play											0.01	(−0.115,0.130)
F	16.56^***^	15.97^***^	15.98^***^	12.42^***^	2.61		1.86					
*R*^2^	0.24	0.43	0.21	0.24	0.05	0.11						
adj *R*^2^	0.22	0.41	0.20	0.22	0.03	0.05						
*R*^2^-change		0.20		0.03		0.06						

* *p < 0.05*;

** *p < 0.01*;

*** *p < 0.001; 95% CI*.

In Hungary, we also performed hierarchical regression analyses to determine the extent to which parental play beliefs and preschoolers home experiences predict children's EF skills. Since sociodemographic variables (preschoolers' age and SES) were significantly related to EF skills that includes inhibitory control, shifting, and VSWM in the present study, these variables were entered as a control into the regression equation. Based on the results from bivariate correlation, the additional contribution of parental play belief and preschoolers' home activity variables was then examined by simultaneously entering them into the regression equation.

When age and SES were entered simultaneously as predictors of preschoolers' inhibitory control skill, they accounted 18% of the variance [*F*_(2, 103)_ = 11.55, *p* < 0.001]. Then, preschoolers' frequency of breakfast at home, pretend play, peer play, motor play, and parental play support were simultaneously entered into the model. However, frequency of pretend play and parental play support were significant predictors in the model that explained an additional 16% of the variance in preschoolers' inhibitory control score. The final model was significant [*F*_(4, 101)_ = 13.12, *p* < 0.001]. As can be seen in Table 8, the positive beta values of pretend play (β = 0.23, *p* = 0.007) and parental play support (β = 0.31, *p* = 0.001) suggest that preschoolers with frequent engagement in pretend play and parents holding better play support belief predicted better inhibitory control skills of children.

**Table 8:** Summary of hierarchical multiple regression models predicting executive function skills in Hungarian sample.

	**Inhibitory control**				**Visual-spatial working memory**				**Switching**			
	**Step 1**		**Step 2**		**Step 1**		**Step 2**		**Step 1**		**Step 2**	
	**β**	***95% CI***	**β**	***95% CI***	**β**	***95% CI***	**β**	***95% CI***	**β**	***95% CI***	**β**	***95% CI***
Age	0.27	(0.008,0.042)^**^	0.22	(0.005,0.036)^**^	0.33	(0.015,0.052)^**^	0.33	(0.015,0.051)^***^				
SES	0.30	(0.135,0.539)^**^	0.16	(−0.017,0.371)	0.19	(0.006,0.423)^*^	0.18	(−0.002,0.402)	0.22	(0.026,0.360)^*^	0.16	(−0.035,0.308)
Pretend play			0.23	(0.043,0.269)^**^								
Play support			0.31	(0.025,0.086)^**^							0.22	(0.003,0.056)^*^
Fine motor							0.25	(0.062,0.370)^**^				
F	11.55^***^	13.12^***^	9.93^***^	9.66^***^	5.27^*^		5.21^**^					
*R*^2^	0.18	0.34	0.17	0.23	0.05	0.09						
adj *R*^2^	0.17	0.32	0.15	0.20	0.04	0.08						
*R*^2^-change		0.16		0.06			0.04					

* *p < 0.05*;

** *p < 0.01*;

*** *p < 0.001; 95% CI*.

Again, in the Hungarian sample, age and SES together explained 17% of the variance in VSWM [*F*_(2, 100)_ = 9.93, *p* < 0.001]. Next, preschoolers' frequency of participation in pretend play, fine motor activities, and arts and crafts were simultaneously entered to the equation predicting preschoolers' performance in VSWM task. However, frequency of participation in fine motor activities was the only significant predictor improving the model by 6% and thus, after removing the insignificant predictors from the model the final model explained the total of 23% of the variance in VSWM score [*F*_(3, 99)_ = 9.66, *p* < 0.001]. Lastly, age and SES together entered to the model predicting preschoolers' performance in switching task. However, only SES variable was contributed significantly in model. After removing age, SES explained 5% of the variance in switching score [*F*_(1, 103)_ = 5.27, *p* = 0.024]. Next, based on the bivariate correlation result, pretend play, peer play, sport and physical activities, and parental play support were simultaneously entered into the model. However, only parental play support was found to be significant predictor of switching score that improved the model by 4% [*F*_(2, 102)_ = 5.21, *p* = 0.007].

## Discussion

The study aimed at exploring the cross-cultural variation in the frequency of preschoolers' engagement in home activities and parental beliefs about the role of play in child development and the purpose of preschool education in Ethiopia and Hungary. Further aims of the present investigation were to examine the cross-cultural variation in the development of preschoolers' EF skills including inhibitory control, shifting, and visual-spatial working memory; and to examine the correlations between preschoolers' home experiences (preschoolers' home activities and their parents' play beliefs) and EF cross-culturally. It is important to note that we intended to recruit two samples that did not differ in terms of SES so that any differences between the samples truly reflect cultural variations. In fact, this was confirmed by the non-significant difference between parental educational levels.

We found that Ethiopian parents hold significantly stronger academic focused beliefs than their Hungarian counterparts. This coincided with our finding indicating that Ethiopian preschoolers practiced academic skills after preschool significantly more often than their Hungarian counterparts. However, no significant cross-cultural variation was found on the play support beliefs that parents hold. Ethiopian parents holding stronger academic focused beliefs while holding comparable play support beliefs with their Hungarian counterparts may indicate that Ethiopian parents recognize that play may have some benefits to child development but prefer academic activities over play for their children.

We also found support for our hypothesis regarding the cross-cultural variation of parental beliefs of the primary purpose of preschool education between the two samples. While academic and cognitive development were reported as the primary purposes of preschool education for Ethiopian parents, social-emotional development and enjoyment for children are the principal purposes of preschool education for Hungarian parents. This is in line with the finding reported by Tigistu (2013) indicating that academic practice is the focus of preschool education in Ethiopia to meet the parents' expectations and demands. Also, Metaferia et al. (2020a) reported that Hungarian parents hold the belief that social-emotional development is the primary purposes of preschool education.

Our finding also demonstrated that Ethiopian preschoolers practiced academic skills and arts and crafts activities at their home more frequently than their Hungarian counterparts. In contrast, our finding depicted that Hungarian preschoolers more frequently engage in fine-motor activities, solitary play, and sports and physical activities at their home compared to their Ethiopian counterparts. We found comparable frequency of spending mealtime together with family, having breakfast at home, and engage in pretend play, motor play, and peer play between preschoolers in Ethiopia and Hungary.

Our finding indicated that there was no significant cross-cultural variation in preschoolers' performance on any of the EF tasks including the Go/no-go task, the switching task and the Mr. Peanut visual spatial working memory task. Our finding also depicts that, after controlling for age and SES, preschoolers' frequency of participation in pretend play and their parents' play support are important variables predicting their inhibitory control skills in both the Ethiopian and the Hungarian samples. These results indicate that preschoolers with parents acknowledging the importance of play for the overall development of children and frequently engage in pretend play have better inhibitory control skills than their counterparts. This finding is consistent with the findings reporting that parental play support (Metaferia et al., 2020a,b) and children's engagement in pretend play (Kelly and Hammond, 2011; Metaferia et al., 2020a) are important factors associated with the development of inhibitory control.

The importance of parental play support for the development of inhibitory control in preschoolers could be explained by different possible means. For instance, parents with strong play support beliefs could better facilitate children's play by different means ranging from supplying play resources to their children to actively engaging in their children's play activities (Johnson et al., 2005) that could in turn improve the parent-child interaction, parental scaffolding, and intimacy. Several studies (see Bernier et al., 2010, 2012; Hammond et al., 2012; Fay-Stammbach et al., 2014) underline the positive contributions of these variables in children's inhibitory control skills.

Our result also demonstrated a cross-cultural variation in the role of breakfast in preschoolers' inhibitory control; on top of parental play support and frequency of pretend play, frequency of breakfast at home proved to be an important factor predicting the development of inhibitory control skills in the Ethiopian sample only. This result replicated the finding reported by Metaferia et al. (2020b) indicating that frequency of breakfast at home is an important factor associated with preschoolers' performance in inhibitory control task. On top of that, the correlation between preschoolers' experience of breakfast at home and their inhibitory control skill is significantly higher in the Ethiopian sample than in the Hungarian.

As expected, we found a cross-cultural variation in preschoolers' home experiences associated with the development of VSWM and shifting skills. While frequency of participation in arts and crafts activities was the important factor predicting VSWM in Ethiopian preschoolers after accounting for preschoolers' age and family SES, frequency of participation in fine motor activities was important predictor of preschoolers' performance in Mr. Peanut's visual-spatial working memory task for Hungarian preschoolers. We also found an interesting result that parental play support is important predictor of the shifting skills in the Hungarian but not in the Ethiopian sample. One of the possible explanations for this cross-cultural variation could be linked to the nature and structure of the play opportunities and activities parents recurrently facilitate for their children at home. Concerning this point, based on their interpretation of the findings from parental belief studies, LaForett and Mendez (2016) underlined that parents' play beliefs could affect the nature of play activities their children frequently engage in. For instance, many of the play activities that Hungarian parents facilitate for their children in their everyday lives could involve the use of shifting skills on top of inhibitory control skills compared to their Ethiopian counterparts.

We believe that the present study makes important contributions to the current literature on the cross-cultural investigation in the development of preschoolers EFs, the role of children's home experience in the development of EF, and the beliefs parents hold about the importance of play in child development and the primary purposes of preschool programs. We focused on samples from two countries that are rarely researched in this field. Yet, it has some limitations. First, while aiming to recruit two samples that are comparable in terms of demographics, both samples were recruited using convenience sampling technique which is not a representative sampling method. Thus, generalization of the findings could be difficult. Besides, it would have been better to use substantially larger samples for a more precise estimation of the results in our cross-cultural investigation. Second, as self-report technique was employed in collecting data from parents about their beliefs of play for child development and the purpose of preschool education and preschoolers' activities at home, the technique might be susceptible to social desirability bias. Third, we have no information regarding the reliability of the measures used to collect preschoolers' home activities as we assessed these variables using individual items. Finally, IQ and language skills of preschoolers, and the effect of other variables within the family and community (such as parenting style, scaffolding, availability of play opportunities including playgrounds) that may be important for the outcome measures were not assessed.

## Conclusion

All in all, from the finding of the present study, we made the following conclusions. While building socio-emotional competence seems to be the primary purpose of preschool education for Hungarian parents of preschoolers, academic and cognitive skills development are the priority for their Ethiopian counterparts. In addition, Ethiopian parents were shown to hold significantly stronger academic focused beliefs than their Hungarian counterparts. However, no significant cross-cultural variation was found between Ethiopian and Hungarian parents on play support beliefs. Ethiopian preschoolers seem to practice academic skills and arts and crafts activities at their home more frequently compared to their Hungarian counterparts. On the other hand, Hungarian preschoolers were reported to engage in fine-motor activities, solitary play, and sports and physical activities more frequently at their home compared to their Ethiopian counterparts. We found no cross-cultural variation in the development of the children's EF skills. Parental play support and preschoolers' participation in pretend play were found to be important variables associated with the development of inhibitory control regardless of the socio-cultural variations between the two nations. However, there were cross-cultural variations in terms of predictive factors associated with the development of both VSWM and shifting skills in preschoolers; while frequent participation in arts and crafts was shown an important home experience for the development of VSWM skills in Ethiopian preschoolers, frequent participation in fine motor activities was found an important factor associated with the development of the same skill in the Hungarian sample. Finally, parental play support seems to be an important element of preschoolers' home experience associated with the development of their shifting skills in Hungarian, but not in Ethiopian preschoolers.

## Data Acquisition Statement

The data collection process was started by establishing a rapport with preschool directors. Consent papers were sent to the parents of the preschoolers to request for their participation including their children. When parents return the signed papers to the preschools, they were provided with questionnaire to fill it out and returned it back to the preschools. Trained data collectors administered individual EF tests for the participant children at the preschool centers in a separate and silent rooms. The data collection period ranges from November 2018 to October 2019.

## Data Availability Statement

The raw data supporting the conclusions of this article will be made available by the corresponding author upon request.

## Ethics Statement

The procedure implemented in the current study was approved by the Eötvös Loránd University's Research Ethics Committee (issue number: 2017/209). Written informed consent to participate in this study was provided by the participants' legal guardian/next of kin.

## Author Contributions

BM, JF, and ZT made valuable contributions to conception and design of the study and data analysis and interpretation. BM prepared the manuscript. JF and ZT read the manuscript and provided crucial feedback. All authors approved the submitted version of the manuscript.

## Conflict of Interest

The authors declare that the research was conducted in the absence of any commercial or financial relationships that could be construed as a potential conflict of interest.
