# Supplementary material for: Parents' Views on Play and the Goal of Early Childhood Education in Relation to Children's Home Activity and Executive Functions: A Cross-Cultural Investigation
Source: Front Psychol. 2021 Apr 26;12:646074. doi: 10.3389/fpsyg.2021.646074 (PMC8108989; doi:10.3389/fpsyg.2021.646074)
Supplement: Supplementary file 1 [file Data_Sheet_1.PDF]

Appendix: Comparison of correlations from the two samples

(done using online calculator at https://www.psychometrica.de/correlation.html#independent)

Variable	Country/sample		Correlation in Ethiopian sample	Correlation in Hungarian sample	Test Statistic *z*	Probability *p*
	Ethiopia Correlates	Hungary Correlates				
VSWM	Pretend	Pretend	.25** N=137	19* N=118	0.496	0.31
	Fine motor	Fine motor	.19* N=138	.23* N=118	-0.33	0.371
	Arts and crafts	Arts and crafts	.19* N=134	.19* N=118	0	0.5
Inhibition	Breakfast	Breakfast	.42** N=132	.20* N=122	1.927	0.027
	Pretend	Pretend	.41** N=133	.33** N=124	0.735	0.231
	Peer paly	Peer play	.29** N=130	.21* N=123	0.671	0.251
	Paly support	Play support	.54** N=126	.40** N=120	1.398	0.081
Switching	Pretend	Pretend	.20* N=123	.19* N=121	0.08	0.468
	Play support	Play support	.23* N=117	.23* N=118	0	0.5
	peer play	Peer play	18* N=121	.22* N=120	-0.319	0.375
